# Fibrous Dysplasia Masquerading as Sternal Malignancy: A Rare and Challenging Presentation

**DOI:** 10.7759/cureus.50833

**Published:** 2023-12-20

**Authors:** Vishal Devarkonda, Shiva Gaddam, Manasa Morisetti, Kshitij Arora, Kavitha Beedupalli

**Affiliations:** 1 Internal Medicine, Louisiana State University Health Sciences Center, Shreveport, USA; 2 Hematology and Medical Oncology, Louisiana State University Health Sciences Center, Shreveport, USA; 3 Pathology and Laboratory Medicine, Louisiana State University Health Sciences Center, Shreveport, USA; 4 Pathology, Louisiana State University Health Sciences Center, Shreveport, USA; 5 Hematology and Oncology, Louisiana State University Health Sciences Center, Shreveport, USA

**Keywords:** albright syndrome, mazabraud syndrome, sternal malignancy, mccune-albright syndrome, fibrous dysplasia (fd)

## Abstract

This case report presents a rare and challenging manifestation of polyostotic fibrous dysplasia (FD), a skeletal developmental anomaly characterized by the proliferation of fibrous connective tissue intermingled with irregular bony trabeculae. While monostotic FD is more common, polyostotic FD can occur in the context of McCune-Albright syndrome, a multisystem developmental disorder. Our patient, a 55-year-old female with a history of diabetes, hypothyroidism, and dyslipidemia, presented with progressively worsening dysphagia, sternal pain, and swelling over three years. Clinical examination revealed a tender and hard swelling in the upper sternal area, prompting further evaluation. Laboratory results, including bone turnover markers, were unremarkable. Imaging studies unveiled a sizable anterior mediastinal lesion with heterogeneous enhancement and coarse calcifications, initially raising concerns of malignancy. Subsequent positron emission tomography scan findings confirmed FD involvement in both the sternum and right femur. Histopathology of the mediastinal mass revealed a spindle cell neoplasm with bony metaplasia, consistent with FD, supported by immunohistochemistry. A multidisciplinary team affirmed the diagnosis of polyostotic FD, and follow-up imaging after one year demonstrated no significant change in lesion size, confirming a benign etiology. While bisphosphonate therapy was planned, regrettably, the patient was lost to follow-up. This case underscores the importance of a comprehensive, multidisciplinary approach in diagnosing and managing complex presentations of FD, ultimately contributing to improved patient care and outcomes in such instances.

## Introduction

Fibrous dysplasia (FD) is an uncommon skeletal developmental anomaly characterized by the proliferation of cellular fibrous connective tissue mixed with irregular bony trabeculae. The incidence rate of FD ranges from 1 in 4,000 to 1 in 10,000 [1]. While monostotic FD is relatively common, accounting for up to 7% of all benign bone tumors, polyostotic FD can occur as part of a multisystem developmental disorder called McCune-Albright syndrome (MAS) [2]. FD/MAS affects both sexes and does not show a preference for any specific population. We present a patient who presented with progressive sternal mass, highly concerning for malignancy. This case presents a unique and challenging manifestation of FD, highlighting the need for a multidisciplinary approach to management. By shedding light on this case, we aim to contribute to the existing knowledge on FD and promote a better understanding of this complex disorder.

## Case presentation

A 55-year-old female patient with a medical history of diabetes, hypothyroidism, and dyslipidemia presented to our clinic with dysphagia, pain, and swelling in the upper sternal area and neck, progressively worsening over three years. The pain intensified with neck movement and was rated 7 out of 10 (ranging from 0, indicating no pain, to 10, representing the most severe pain one has ever experienced). The patient described the pain as stabbing, with some relief experienced upon resting. Additionally, she reported decreased appetite and unintentional weight loss, although she could not provide specific measurements.

On physical examination, a 5 cm swelling was observed in the midline above the sternum in the neck region. The swelling was tender to palpation, had non-pulsatile characteristics, had a hard consistency, and did not allow transillumination. Despite these findings, the patient’s vital signs were within normal limits, and further examinations of her cardiovascular, respiratory, abdominal, neurological, and musculoskeletal systems revealed no abnormalities.

Laboratory findings

The patient’s complete blood count and comprehensive metabolic panel were unremarkable, and bone turnover labs were within normal limits (Table 1).

**Table 1:** Bone turnover labs.

Lab	Patient results	High range	Low range	Normal range
C telopeptide (CTX), serum	Within normal limits	>573 pg/mL	<200 pg/mL	200–573 pg/mL
Procollagen type 1 propeptide	Within normal limits	>75 µg/L	<20 µg/L	20–75 µg/L
Alkaline phosphate bone-specific	Within normal limits	>29.0 µg/L	<5.6 µg/L	5.6–29.0 µg/L

Imaging findings

**Figure 1:**
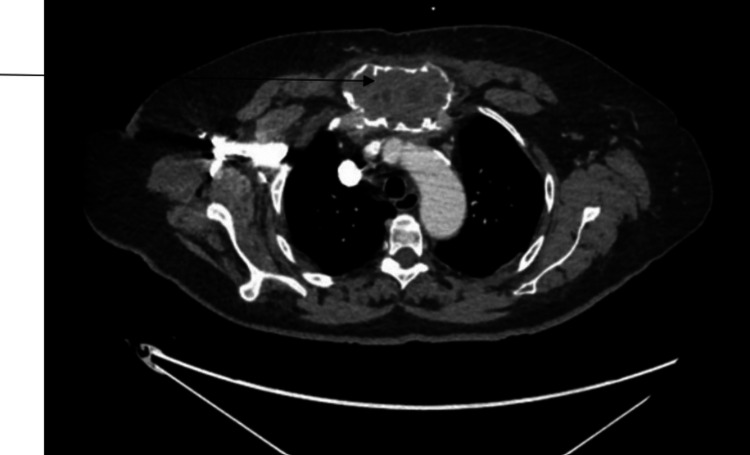
CT of the chest showing the manubrial expansile lesion. The arrow points to the expansile lesion in the CT scan image.

**Figure 2:**
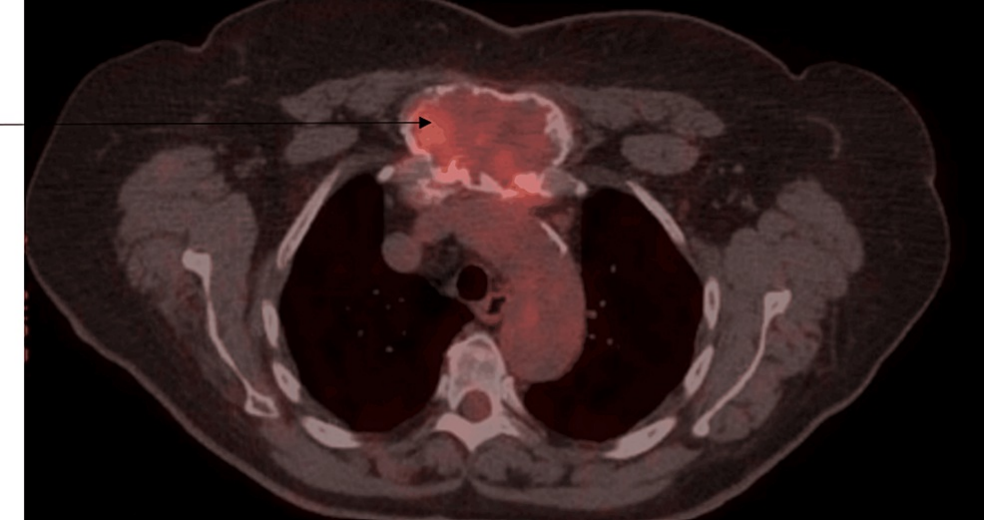
PET/CT showing the manubrial expansible lesion. The arrow points to the increased uptake in the expansile lesion in the manubrium on the PET scan. PET/CT = positron emission tomography/computed tomography

**Figure 3:**
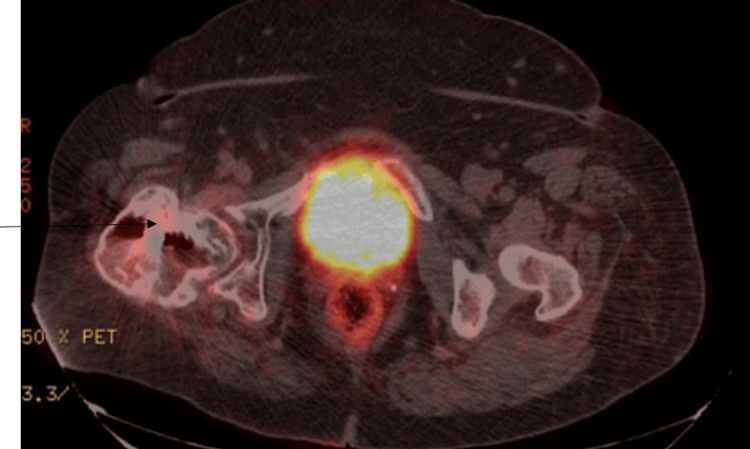
PET/CT showing right-sided femoral expansible lesions. The arrow points to the increased uptake in the right-sided femoral region on the PET scan. PET/CT = positron emission tomography/computed tomography

A CT scan of the neck, chest, abdomen, and pelvis with contrast was performed to evaluate the soft tissues. The scan revealed a large lesion in the anterior mediastinum, displaying heterogeneous enhancement and coarse calcifications at its periphery. The lesion extended anterosuperiorly into the chest wall, measuring approximately 8 x 5 x 7.2 cm (Figure 1). This was concerning for malignancy. A positron emission tomography (PET) scan was done for further diagnostic purposes, which showed a right femur expansile lesion measuring at least 8.5 cm in the cephalocaudal dimension, exhibiting increased fluorodeoxyglucose (FDG) activity with a maximum standardized uptake value (SUV) of 4.7, in addition to the known sternal manubrial lesion which on PET measured 4.5 x 7.7 cm and displayed a maximum SUV of 5.8 (Figures 2, 3). These imaging findings at the sternum and the femur were similar.

Clinical pathology

**Figure 4:**
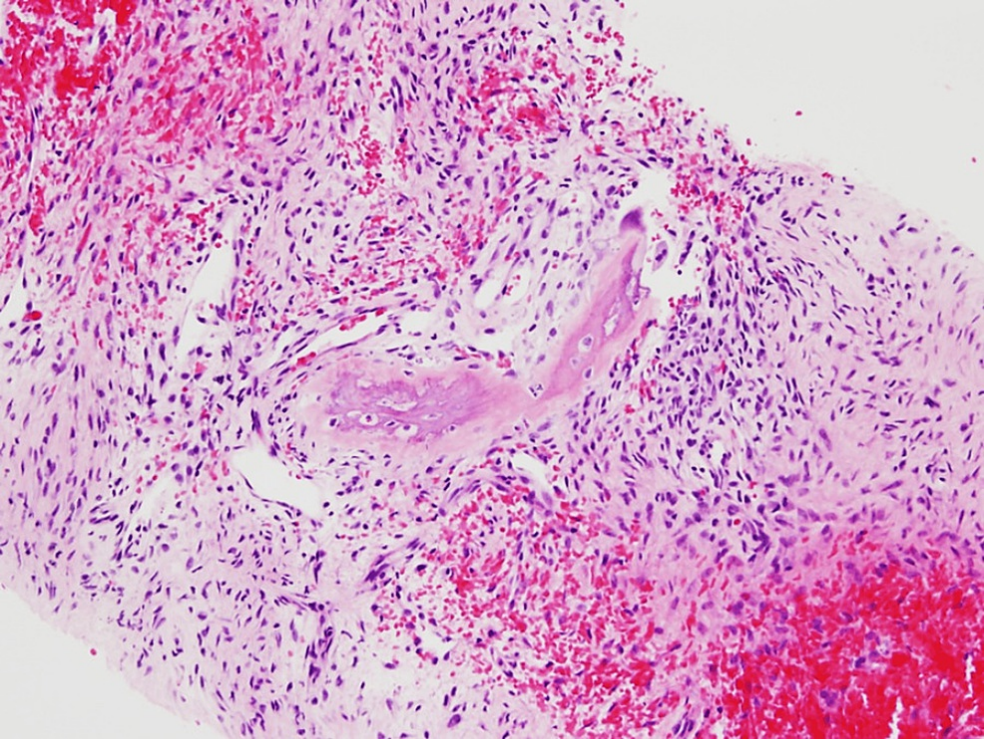
Pathology of the manubrium mass showing woven bone collagen involving the dense stromal fibers (10× magnification).

**Figure 5:**
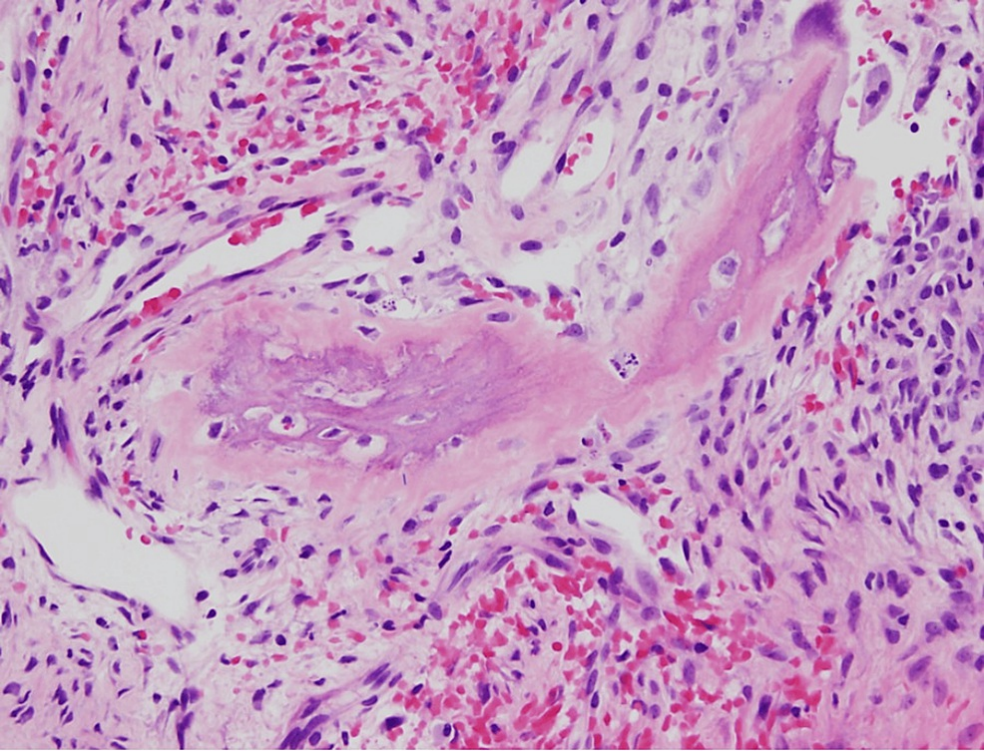
Pathology from the manubrium site showing woven bone collagen encroaching into the dense stromal fibers (sharpie fibers; 20× magnification).

**Figure 6:**
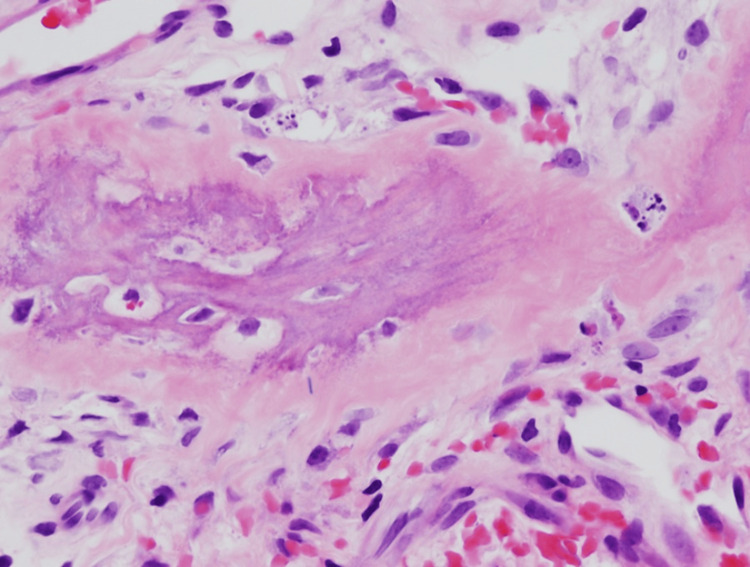
Pathology from the manubrium site showing woven bone collagen encroaching into the dense stromal fibers (sharpie fibers; 40× magnification).

The initial pathology from the mediastinal mass revealed a spindle cell neoplasm with bony metaplasia. Scattered osteoclast-type giant cells and abundant hemorrhage were observed. Additionally, a focal area of bone formation was identified, and no atypical osteocytes or cartilage were present. The spindle cells appeared bland without significant atypia. No significant mitoses, necrosis, lymphocytes, plasma cells, or myxoid components were detected. The absence of lymphoid aggregates or lymphocytes made lymphoma unlikely (Figures 4-6). Immunohistochemistry was performed using CD34, STAT6, SMA, S100, and Ki-67 markers. The spindle cells tested negative for CD34 and STAT6, making a solitary fibrous tumor unlikely. CD34 staining was observed in blood vessels, serving as an internal control. SMA and S100 were also negative, suggesting that smooth muscle tumor, vascular neoplasm, and neuronal neoplasm were unlikely. However, Ki-67 staining revealed a mild increase in Ki-67-positive cells.

The differential diagnosis at this stage included (1) a benign or malignant spindle cell neoplasm, (2) a portion of teratoma, (3) a bone-forming neoplasm with giant cells, and (4) FD.

Clinical course

**Figure 7:**
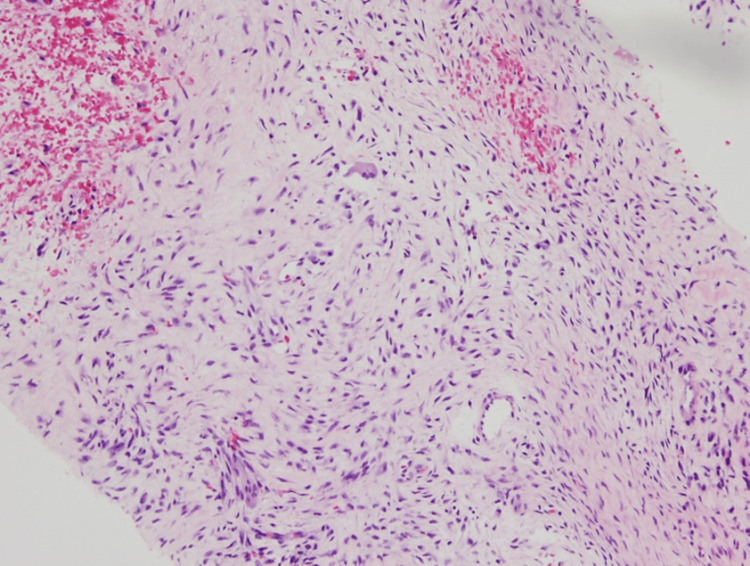
Pathology from the femoral site showing haphazardly arranged stromal fibers and multinucleated giant cells (10× magnification).

**Figure 8:**
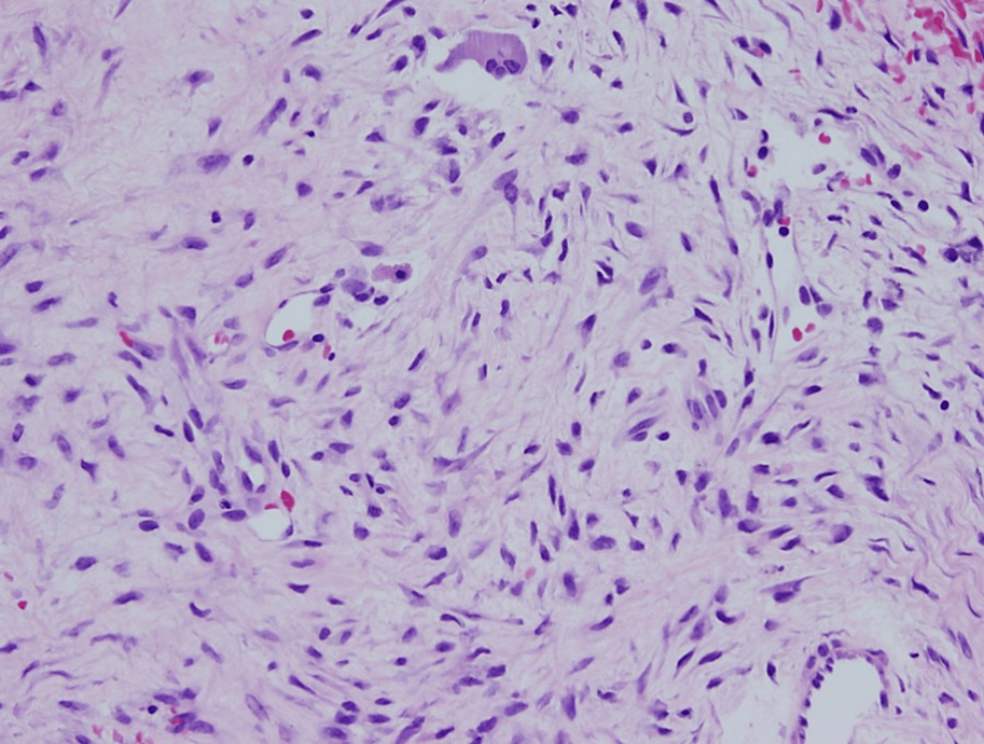
Pathology from the femoral site showing haphazardly arranged stromal fibers and multinucleated giant cells (20× magnification).

The follow-up pathology involved a needle core biopsy of the right femur mass, which showed scant hypocellular fibrous tissue without atypia; it was determined to be similar to the sternal bone lesion (Figures 7, 8). A multidisciplinary team involving endocrinology, radiology, oncology, and pathology was involved in the case. Although there was no cortical destruction, bone remodeling was observed. These radiological findings were suspected to be related to a benign entity, including polyostotic fibrodysplasia. The presence of scant hypocellular fibrous tissue without atypia on pathology and in consultation with endocrinology confirmed the diagnosis of polyostotic fibrodysplasia. A repeat CT done after one year showed similar findings with no further increase in the size of the lesion, confirming a benign etiology. The multidisciplinary team planned to initiate treatment with bisphosphonates to address the patient’s symptoms and manage the condition. However, unfortunately, the patient was lost to follow-up, and no further management was possible. The collaborative effort of the radiology, pathology, and endocrinology teams was crucial in reaching the diagnosis and formulating the treatment plan for the patient.

## Discussion

FD is characterized by replacing portions of the bone with fibrous connective tissue and inadequately formed trabecular bone [3]. This process typically begins in the medullary cavity and results from a postzygotic mutation in the *GNAS1 *gene rather than being a true neoplasm. While FD is considered more of a skeletal dysplasia, it still constitutes roughly 5-7% of all benign bone tumors [4].

FD can occur in either a single bone (monostotic) or multiple bones (polyostotic), with the polyostotic form usually presenting in childhood. It can be associated with MAS (also called Albright syndrome). In addition to FD, this syndrome is characterized by café-au-lait macules with jagged borders and endocrine abnormalities, such as excessive growth hormone, to which the affected bone is more sensitive than the unaffected bone [5]. Patients with polyostotic lesions may also present with soft tissue myxomas, known as Mazabraud syndrome [6]. The clinical presentation of patients with MAS can vary depending on the disease features, with various endocrine diseases potentially presenting due to increased hormone production. These include precocious puberty, testicular abnormalities, hyperthyroidism, excess growth hormone secretion, Cushing’s syndrome (rare), and hypophosphatemia, which may lead to rickets, osteomalacia, and worsened skeletal outcomes [5].

FD typically manifests in individuals in their teens or 20s. While it can occur in any bone, it is most commonly found in the proximal femur, tibia, ribs, and skull. The condition affects slightly more males than females [7]. Although most patients with FD do not experience symptoms, it may cause pain or swelling and, in some cases, repeated pathologic fractures or severe bone deformities such as the “shepherd’s crook” varus deformity of the proximal femur [7]. Malignant transformation is rare, occurring in less than 1% of patients. Hence, these patients should be followed up for clinical changes [8].

FD can be visualized on radiographs as a lytic lesion in the metaphysis or diaphysis, exhibiting a ground-glass matrix with potential bowing and bone expansion. The cortical bone is thinned and may display endosteal scalloping [9]. The periosteal reaction is typically absent, except in cases where a pathologic fracture has occurred. Although radiological signs are diagnostic in most cases, some cases with atypical findings may warrant biopsy for histological evaluation for malignancy. The presence of symptoms determines the approach to treating FD. The frequency of follow-up radiographs and their need depend on the site of involvement and other clinical features. In cases where FD is found in weight-bearing bones such as the proximal femur, it may lead to aggressive remodeling and fractures, necessitating regular monitoring through serial radiographs [10]. The burden of FD can be effectively evaluated using 18F‐NaF‐PET/CT, enabling precise assessment of FD activity and establishing quantitative correlations with clinically relevant skeletal outcomes [11]. Bone turnover markers (BTMs), including alkaline phosphatase, procollagen 1 intact N-terminal propeptide, and C-terminal telopeptide, are commonly utilized as indicators of disease activity. However, it is essential to consider that serum levels of these markers can be affected by factors such as age, comorbidities, and treatments, including bisphosphonates and denosumab [12].

FD associated with symptoms such as pain, deformity, or fractures may require curettage, bone grafting, and stabilization. However, there is a high likelihood of recurrence after surgery. Autografts should be avoided as they tend to be resorbed. For symptomatic patients, bisphosphonate therapy is another treatment option [12]. Interestingly, Florenzano et al. investigated the natural history of FD disease activity, its association with pain, and the impact of bisphosphonates on BTMs and FD burden progression in childhood. They found that BTMs decline with age, but bisphosphonate treatment does not significantly affect this decline. Pain is more severe in adults but not related to BTMs. Bisphosphonates do not prevent FD burden progression in children. These findings should be considered when using bisphosphonates in FD treatment and BTMs as surrogate endpoints [13].

## Conclusions

The patient exhibited a gradually progressive sternal mass, an atypical presentation of FD. Collaboration among the medical team led to the confirmation of the diagnosis. This case report presents a rare and challenging manifestation of polyostotic FD, emphasizing the need for a thorough diagnostic approach and multidisciplinary management. This report contributes valuable insights to the understanding and treatment of this complex condition, guiding improved patient care and outcomes.
